# Transcriptome-Based Discovery of *Fusarium graminearum* Stress Responses to FgHV1 Infection

**DOI:** 10.3390/ijms17111922

**Published:** 2016-11-17

**Authors:** Shuangchao Wang, Jingze Zhang, Pengfei Li, Dewen Qiu, Lihua Guo

**Affiliations:** 1 State Key Laboratory for Biology of Plant Disease and Insect Pests, Institute of Plant Protection, Chinese Academy of Agricultural Science, Beijing 100081, China; yuxinren2006@163.com (S.W.); jingzezhang0820@gmail.com (J.Z.); li_pengfei2014@163.com (P.L.); qiudewen@caas.cn (D.Q.); 2 Walloon Centre of Industrial Biology, Gembloux Agro-Bio Tech, University of Liège, Passage des Déportés, 2, Gembloux 5030, Belgium

**Keywords:** *Fusarium graminearum*, Fusarium graminearum hypovirus 1, transcriptome, stress responses, cellular redox regulation

## Abstract

Fusarium graminearum hypovirus 1 (FgHV1), which is phylogenetically related to Cryphonectria hypovirus 1 (CHV1), is a virus in the family *Hypoviridae* that infects the plant pathogenic fungus *F. graminearum*. Although hypovirus FgHV1 infection does not attenuate the virulence of the host (hypovirulence), it results in defects in mycelial growth and spore production. We now report that the vertical transmission rate of FgHV1 through asexual spores reached 100%. Using RNA deep sequencing, we performed genome-wide expression analysis to reveal phenotype-related genes with expression changes in response to FgHV1 infection. A total of 378 genes were differentially expressed, suggesting that hypovirus infection causes a significant alteration of fungal gene expression. Nearly two times as many genes were up-regulated as were down-regulated. A differentially expressed gene enrichment analysis identified a number of important pathways. Metabolic processes, the ubiquitination system, and especially cellular redox regulation were the most affected categories in *F. graminearum* challenged with FgHV1. The p20, encoded by FgHV1 could induce H_2_O_2_ accumulation and hypersensitive response in *Nicotiana benthamiana* leaves. Moreover, hypovirus FgHV1 may regulate transcription factors and trigger the RNA silencing pathway in *F. graminearum*.

## 1. Introduction

Fungal viruses, which are referred to as mycoviruses, have been discovered throughout the kingdom of fungi. Most mycoviruses develop a co-existent relationship with their host. In many cases, mycovirus infection is not associated with host phenotypic changes (asymptomatic infections). Interestingly, the capacity of the fungus *Curvularia protuberata* to confer heat tolerance to its host plant, *Dichanthelium lanuginosum*, is related to the presence of the mycovirus Curvularia thermal tolerance virus (CThTV) in a three-way symbiosis that is required for thermal tolerance [1]. However, some mycoviruses, such as Cryphonectria parasitica hypovirus 1 (CHV1) and Fusarium graminearum virus 1 (FgV1), cause severe phenotypic alterations, including inhibition of sexual development, defects in pigment production, and reductions in growth rates and virulence [2,3]. Mycoviruses are attracting attention in the study of fundamental fungal cellular processes due to their critical effects and the interactions between pathogenic fungi and viruses.

*F. graminearum* causes a destructive disease called *Fusarium* head blight in wheat and other cereal grains. In addition to reduced grain yield, *Fusarium* species-infected crops are contaminated with mycotoxins, such as trichothecenes and zearalenone, which make the infected grain toxic to human and animal consumers [4]. In addition, the genome of the *F. graminearum* strain PH-1 was sequenced and published in 2003 by the Whitehead Institute, Center for Genome Research (Cambridge, MA, USA). It contains a total of 13,322 genes with a length of 17,842,161 bp and will provide the fundamental genetic information necessary for pathogenicity studies [5]. Several mycoviruses, including FgV1, F. graminearum virus 2 (FgV2), F. graminearum virus 3 (FgV3), F. graminearum virus 4 (FgV4), F. graminearum hypovirus 1 (FgHV1), F. graminearum hypovirus 2 (FgHV2), Fusarium graminearum mycotymovirus 1 (FgMTV1), Fusarium poae dsRNA virus 2 (FpV2), Fusarium poae dsRNA virus 3 (FpV3), and Fusarium graminearum deltaflexivirus 1 (FgDFV1) have been identified from isolates of *F. graminearum* [3,6,7,8,9,10,11].

Infection with some hypoviruses affects fungal virulence or other phenotypes, including colony morphology, sporulation, and growth rates [12]. Previous studies have demonstrated that host fungal virulence is dramatically reduced during hypovirus CHV1-EP713 and CHV2-NB58 infections [2,13]. FgHV1 is a member of the *Hypoviridae* family, a group of positive-strand RNA viruses. Although FgHV1 is closely related to CHV1 and CHV2, the virulence of *F. graminearum* is not impacted by FgHV1 infection. But FgHV1 infection caused reduction of growth rates and spore production [7]. These joint effects of FgHV1 must be important for its co-existent relationship with *F. graminearum*.

Hypovirus-infected *C. parasitica* serves as a good model for exploring changes in transcript accumulation during fungi-virus interactions. Gene expression differences between hypovirus-infected *C. parasitica* and an isogenic virus-free strain have been examined using mRNA differential display technology [14]. With a robust microarray platform for *C. parasitica*, comparisons of transcript accumulation in hosts infected with the severe hypovirus CHV1-EP713 and the mild CHV1-Euro7, both of which belong to the family *Hypoviridae*, were also performed [15]. Although CHV1-Euro7 shares a high level of sequence identity at both the nucleotide (87% to 93%) and the amino acid (90% to 98%) levels with CHV1-EP713, significant differences in the *C. parasitica* transcriptome were observed. Based on the application of next-generation sequencing technology, Lee et al. recently reported transcriptional changes following the infection of *F. graminearum* with four mycoviruses from four different families [16]. There has been extensive research on the gene expression changes that occur in *C. parasitica* infected with hypoviruses such as CHV1/EP713 and CHV1-Euro7 compared with the wild-type strain. However, there are no reports on the changes in transcript accumulation in hypovirus-infected *F. graminearum*. To address this deficiency, we performed a genome-wide transcriptome analysis of *F. graminearum* in response to FgHV1 infection using RNA-seq to identify differentially expressed genes. The transcriptome analysis of virus-infected fungi will help elucidate the genes regulated by hypovirus infection that are involved in growth, development, and stress responses and direct further studies into the interactions between pathogenic fungi and viruses.

## 2. Results

### 2.1. FgHV1 Was Transmitted to All F. graminearum Asexual Spores

**Figure 1:**
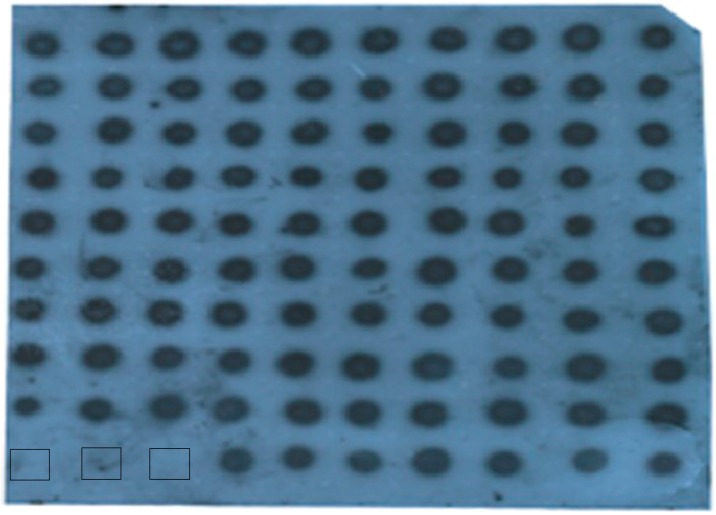
Detection of viral dsRNA using Northern dot blots. The first four samples in the last row, from left to right, correspond to blank control, *F. graminearum* PH-1, *F. graminearum* HN10-11F, and *F. graminearum HN10*. All other samples were derived from spores of *F. graminearum* HN10.

Mycoviruses are transmitted in two ways, horizontal and vertical transmission. Vertical transmission by sporulation is a primary means of mycovirus spread. As we described previously, there was about a 28% reduction in conidia production resulting from the FgHV1-infection. Asexual spores are produced from modified fungi hyphae whose growth rate was slightly reduced by FgHV1. Based on the above-mentioned factors, we are quite curious about whether FgHV1 can be transmitted to the asexual spores through the cytoplasm as the spores develop. To assay vertical transmission of the virus through the conidia, we tested more than 96 conidia for the presence of virus using northern dot blots with FgHV1 genome-specific digoxigenin (DIG)-labeled probes as we described. As Figure 1 showed, dot blot hybridization indicated that all conidia tested were infected with FgHV1. Although FgHV1 caused asexual spore production reduction, asexual spores still acted as a primary means for FgHV1 transmission in *F. graminearum*.

### 2.2. Illumina RNA-Seq and Overall Transcriptional Profiles in F. graminearum in the Presence of FgHV1

**Figure 2:**
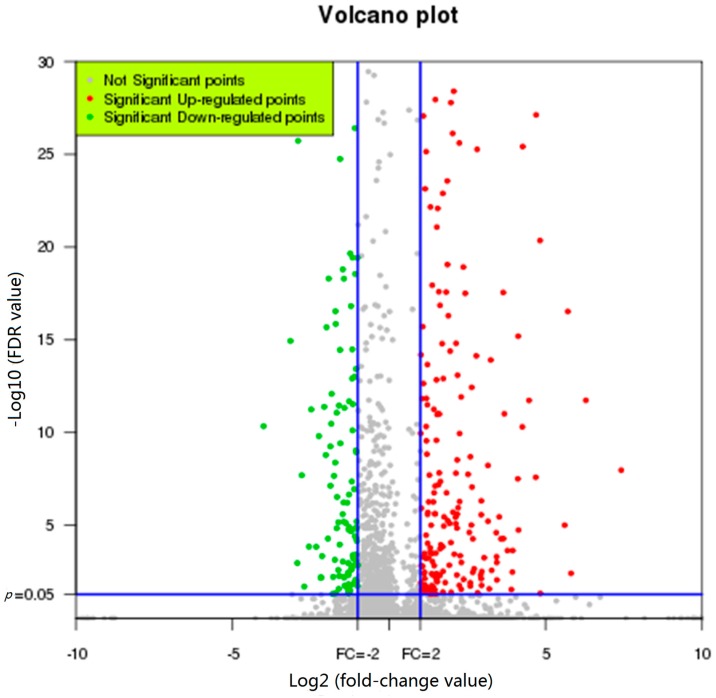
Volcano plot displaying differentially expressed genes between virus-infected and virus-free samples. The *y*-axis displays to the -log10 of the mean expression value (FDR value), and the *x*-axis displays the Log2 (fold-change (FC)-value). The red dots represent the up-regulated differentially expressed transcripts between virus-infected and virus-free samples; the green dots represent the transcripts whose expression was down-regulated; and the gray dots indicate genes not showing significant differences.

To elucidatee the molecular networks that caused the phenotypic alterations in *F. graminearum*, the transcriptional response of *F. graminearum* to hypovirus FgHV1 infection was examined using RNA-seq. We harvested mycelia from two isogenic strains after four days of culture and extracted total RNA. cDNA libraries were constructed and sequenced using the Illumina HiSeq™ 2000 platform (BGI, Shenzhen, China), as described in the Materials and Methods. On average, 53,577,329 Illumina raw reads were generated for each sample (Table 1). After removing adaptor sequences, ambiguous nucleotides, and low-quality sequences, an average of 49,940,923 clean reads were obtained for every sample. The obtained reads were mapped to the reference sequence of the *F. graminearum* strain PH-1 using SOAPaligner/SOAP2. The genome mapping rates for reads from the virus-infected and virus-free libraries were 84% and 83%, respectively, on average. We also aligned the reads to the FgHV1 genome and assembled the reads into transcripts using TopHat and Cufflinks. Differentially expressed genes (DEGs) were identified using Cuffdiff implemented in Cufflinks. The Fragments Per Kilobase of exon per Million reads mapped (FPKMs) were used to calculate the expression level of each gene. The resulting Pearson’s correlation coefficients (*R* = 0.97) between the replicates for virus-infected and virus-free samples were significant (Figure S1). Differential expression analysis was first performed based on a two-fold change threshold for expression relative to the virus-free sample and a false discovery rate (FDR) < 0.05 or a *p*-value < 0.05. A total of 378 genes were differentially expressed (Table S1). In this study, DEGs showing higher expression levels in virus-infected samples (Group 1) than in virus-free samples (Group 2) were considered ‘up-regulated’, while those with lower expression levels in Group 1 were considered ‘down-regulated’. As shown in Figure 2, among these DEGs, 248 genes were up-regulated, and 130 genes were down-regulated. In addition, the FgHV1-encoded ORF A and ORF B transcripts were highly expressed in the FgHV1-infected *F. graminearum* strain, whereas no mRNAs from either ORF A or ORF B were detected in the virus-free strain. This result also confirmed the absence of FgHV1 in the virus-free strain HN10-11F.

### 2.3. Representative Transcripts with Significant Changes in Expression in Response to FgHV1 Infection

To determine the mostly highly affected transcripts, the top 20 downre-gulated and up-regulated genes with the highest fold changes were analyzed (Table 2). We searched the *Fusarium* comparative database and the NCBI database for these genes. Unfortunately, not all of these genes were annotated, and most of them encoded hypothetical proteins with conserved domains. Among these genes, several associated with the fungal trichothecene efflux pump (TRI12), alcohol dehydrogenase, cytochrome P450 oxidoreductase, the major facilitator superfamily (MFS) transporter, and the ATP-binding cassette (ABC) transporters were strongly down-regulated. Genes encoding glutathione-dependent formaldehyde-activating enzyme, endoribonuclease L-PSP, and an SRR1 family member were strongly activated. Lee et al. identified 12 common DEGs in *F. graminearum* following infection with four mycoviruses, FgV1, FgV2, FgV3 and FgV4 [16]. Among these genes, *FGSG_00878* and *FGSG_07582* were also differentially expressed in the presence of FgHV1, showing down-regulation. *FGSG_00878* encodes a hypothetical protein of unknown function, while *FGSG_07582* belongs to the MFS. The MFS transporters are single-polypeptide secondary carriers capable of transporting small solutes and might be involved in mycovirus recognition and the stress response in *F. graminearum*.

### 2.4. Gene Ontology (GO) Analysis of DEGs

**Figure 3:**
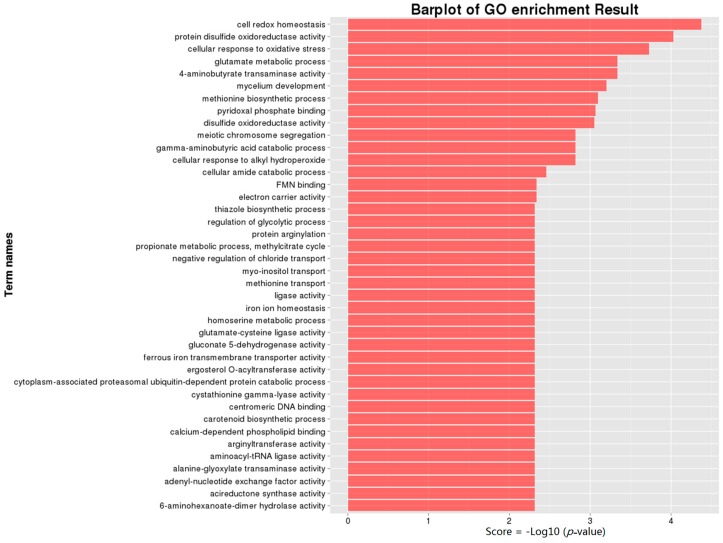
GO ontology classifications of differentially expressed genes. 305 DEGs were subjected to GO annotation. The vertical axis shows the names of clusters of GO terms, and the horizontal axis displays the −Log10 (*p*-value). A *p*-value < 0.05 was used as a threshold to select significant GO terms.

GO analysis is a strictly defined concept that is used widely in functional annotation and enrichment analysis in all types of organisms. To gain insights into the functions of the genes regulated by FgHV1 infection, we analyzed the DEGs for enriched GO terms. The identified GO terms were classified into three major functional ontologies (biological process, cellular component, and molecular function) and are all listed in the additional file Table S2. As shown in Figure 3, cell redox homeostasis (GO:0045454), protein disulfide oxidoreductase activity (GO:0015035), and the cellular response to oxidative stress (GO:0034599) were the mostly highly enriched GO terms. All three of these GO terms are involved in the oxidative stress response. In addition, terms such as disulfide oxidoreductase activity (GO:0015036), oxidoreductase activity acting on the CH-NH2 group of donors (GO:0016638), monophenol monooxygenase activity (GO:0004503), glutathione peroxidase activity (GO:0004602), and glutathione transferase activity (GO:0004364) were enriched. Transcripts involved in the carotenoid biosynthetic process (GO:0016117) were down-regulated. A set of GO terms associated with mycelial growth and development was also enriched, including mycelium development (GO:0043581), glutamate metabolic process (GO:0006536), regulation of glycolytic process (GO:0006110), negative regulation of gluconeogenesis (GO:0045721), glycerol metabolic process (GO:0006071), and filamentous growth (GO:0030447). In particular, the conidiophore development (GO:0070787) was enriched. Moreover, transcripts involved in transportation were enriched in GO terms such as FMN binding (GO:0010181) and negative regulation of chloride transport (GO:2001226).

### 2.5. Kyoto Encyclopedia of Genes and Genomes Pathway (KEGG) Annotation of DEGs

**Figure 4:**
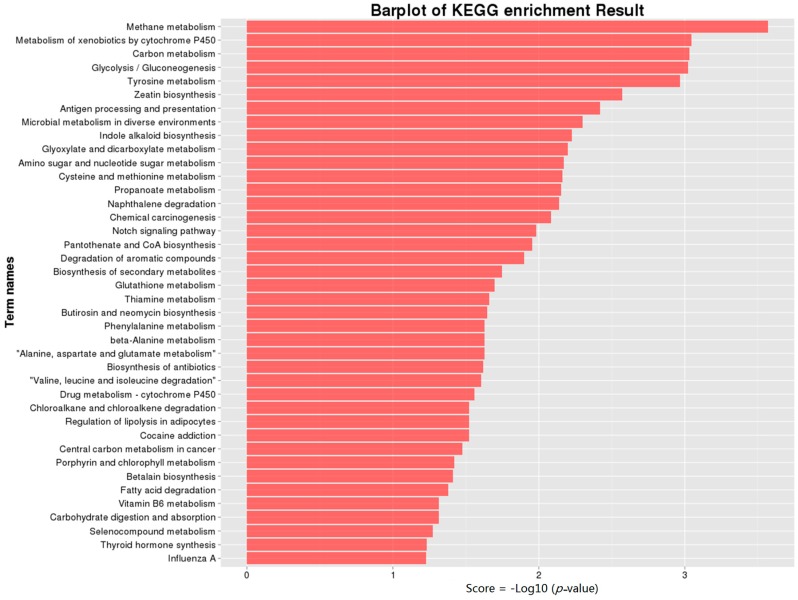
KEGG annotation of differentially expressed genes. 78 DEGs were subjected to KEGG annotation and were grouped into 37 pathway classes. The vertical axis shows the name of KEGG terms and the horizontal axis displays the −Log10 (*p*-value). A *p*-value < 0.05 was used as a threshold to select significant KEGG pathways.

KEGG pathway analysis can reveal specific pathways that are induced or suppressed by virus infection. To understand the interactions of genes with various biological functions, we conducted searches against the KEGG using BLASTx. The search results indicated that 117 KEGG pathways were mapped (Table S3). As shown in Figure 4, Methane metabolism (ko00680), Metabolism of xenobiotics by cytochrome P450 (ko00980) and Carbon metabolism (ko01200) were the top three enriched pathways. Pathways, including glycolysis/gluconeogenesis (ko00010), alanine, aspartate and glutamate metabolism (ko00250), regulation of lipolysis in adipocytes (ko04923), and fatty acid degradation (ko00071) that may influence the mycelial growth rate were enriched. Although the red *Fusarium* pigment was not affected by FgHV1 infection, FGSG_03181, categorized into the Betalain biosynthesis (ko00965) pathway, was up-regulated in the FgHV1-infected *F. graminearum* HN10-11F strain. It is worth noting that pathways including the metabolism of xenobiotics by cytochrome P450 (ko00980), drug metabolism-cytochrome P450 (ko00982), and glutathione metabolism (ko00480) pathways were consistent with the results of the GO analysis related to the biological process of cellular redox regulation, as discussed above.

Viruses require the host translational machinery to produce viral proteins for proliferation. In our GO and KEGG analysis, many items associated with protein synthesis and processing were identified. tRNA aminoacylation for protein translation (GO:0006418), regulation of ribosome biogenesis (GO:0090069), and protein processing in the endoplasmic reticulum (ko04141) were enriched. Through the addition of one ubiquitin molecule (monoubiquitination) or a chain of ubiquitin molecules (polyubiquitination) to a substrate protein, the ubiquitination system functions in a wide variety of cellular processes [17]. Many ubiquitin-related GO terms and pathways were identified, including cytoplasm-associated proteasomal ubiquitin-dependent protein catabolic processes, protein denaturation involved in proteasomal ubiquitin-dependent protein catabolism, and negative regulation of ubiquitin-specific protease activity.

### 2.6. Induction of H_2_O_2_ Accumulation and Hypersensitive Response by FgHV1

**Figure 5:**
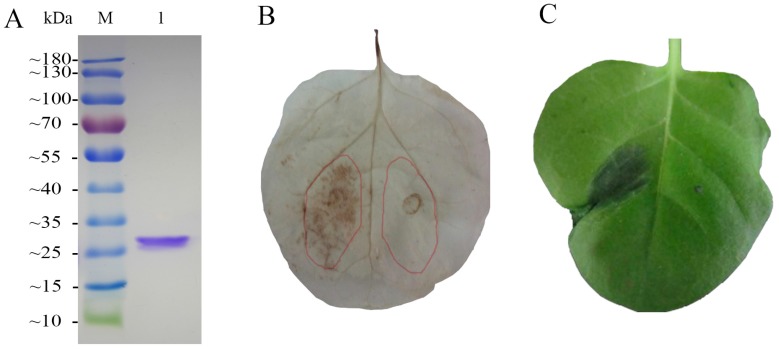
H_2_O_2_ accumulation and HR in tobacco following p20 treatment. (**A**) Purification of recombinant p20. The proteins were resolved on SDS-PAGE with Coomassie brilliant blue R-250 staining. **Lane M**, protein molecular mass marker; **Lane 1**, p20; (**B**) Observation of H_2_O_2_ accumulation in *N. benthamiana* leaves. The left side of the leaf was injected with p20 and the other side was injected with BSA as a negative control. The infiltration extent was drawn onto the leaf right after infiltration and marked in the picture; (**C**) Hypersensitive response induced by p20 in *N. benthamiana*. P20 (left side) and Tris-HCL (right side) were injected into same leaf. The hypersensitive response was observed at 24 h postinjection.

Through our analysis of DEGs enrichment, it was found that oxidative stress reaction may be a primary cellular response during the FgHV1 infection. Many DEGs, GO terms, and KEGG pathways at the top were related to redox status regulation. Reactive oxygen species, especially H_2_O_2_ serve as critical oxidative stress signaling molecules and are important in plant defense reaction. The p20, encoded by FgHV1 was a papain-like proteinase which caught our attention and was purified (Figure 5A). As shown in Figure 5B, hydrogen peroxide polymerized by diaminobenzidine (DAB), which forms a dark red-brown precipitate, was detected in p20 treated *N. benthamiana* leaves. This phenomenon indicated that the recombinant p20 could induce H_2_O_2_ accumulation. As we know, H_2_O_2_ may lead to hypersensitive response. Therefore, we also examined whether p20 could induce hypersensitive response in *N. benthamiana* leaves. After 24 h, the purified recombinant p20 can also induce a typical hypersensitive response in *N. benthamiana* (Figure 5C).

### 2.7. Quantitative Real-Time Reverse Transcription PCR Validation of the RNA-Seq Data

**Figure 6:**
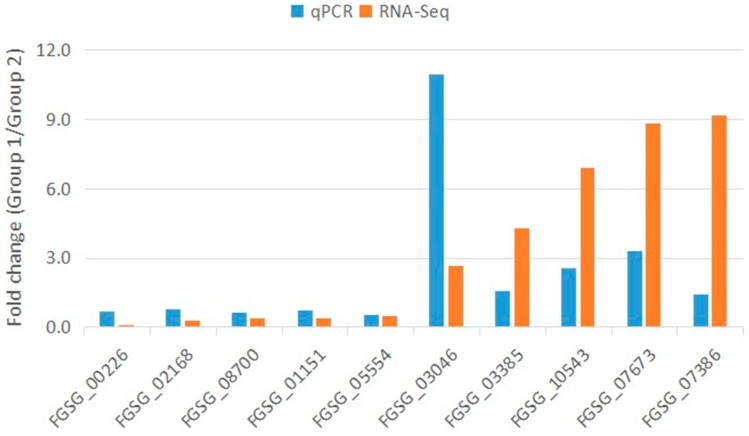
qRT-PCR validation of RNA-seq data. The expression of 10 selected genes was examined via real-time RT-PCR. For each qRT-PCR validation, three technical replications were performed. The beta-tubulin gene was used as an internal control.

As a highly sensitive technique, qRT-PCR provides a reliable method to detect transcripts of genes of interest, especially when gene expression is very low. To confirm the RNA-seq results, we picked 10 representative genes to perform qRT-PCR. The β-tubulin gene (*FGSG_09530*) was used as an endogenous control for normalization. All of the primers designed using Beacon designer V8.12 (Premier Biosoft, Palo Alto, CA, USA), are listed in additional file Table S4. The qRT-PCR results were mostly consistent with those from the RNA-seq. Although the detected fold changes did not exactly match, most of the genes showed the same trends according to these two methods. For example, the up-regulation of *FGSG_03046* and *FGSG_07673* was confirmed by both RNA-seq and qRT-PCR. As shown in Figure 6, genes that were down-regulated, such as *FGSG_08700* and *FGSG_05554*, also showed decreased levels in the qRT-PCR analysis. However, opposite results were returned for a few genes using the two different methods when we checked some other genes of interest. In addition, while critical RNA silencing-related components were not differentially expressed according to RNA-seq data, we detected changes in these components via the qPCR method (data not published). It is highly likely that qRT-PCR results will not correlate with RNA-seq data when changes in gene expression are very small or when gene expression levels are too low.

## 3. Discussion

Hypovirus infection, which results in a number of distinguishing phenotypic traits, plays an important role in elucidating the interaction between virus and host. Virulence and mycotoxin production of the host *F. graminearum* were not significantly affected [7]. However, FgHV1 infection caused reduced hyphal growth and conidia production. Moreover, the high vertical transmission rate (100%) of FgHV1 through conidia indicated that FgHV1 is well adapted to the host. Phenotypic changes are linked to differential gene expression. In this study, we performed genome-wide expression analysis in response to hypovirus FgHV1 infection. A total of 378 genes were differentially expressed after FgHV1 infection in *F. graminearum*. These data are quite remarkable, as FgHV1 caused fewer phenotypic alterations than other hypoviruses. This study will provide another good example for elucidating host-virus interactions.

Although the hypovirus FgHV1 is simple, it was clear that DEGs resulting from infection with this virus were associated with many biological processes, cellular components, and molecular functions. Metabolic pathways play fundamental roles in hyphal growth and the pathogenic process. Changes in energy metabolism can lead to pleiotropic phenotypic effects, such as alterations in the growth, virulence, and reproduction of the virus-infected host. Among the identified DEGs, a large number of genes were enriched in various metabolic pathways (Figure 3 and Figure 4). The metabolism of carbohydrates, amino acids, and lipids was influenced by FgHV1 infection. Propionate metabolic processes, the methylcitrate cycle and alcohol metabolic processes were up-regulated, while cellular aldehyde metabolic processes and lactate metabolic processes were down-regulated. Additionally, glutamate metabolic processes and homoserine metabolic processes were suppressed, while glycine metabolic process was activated. Down-regulation of these metabolic genes may result in slower mycelial growth and other related phenotypic changes. As we described previously, FgHV1 infection can reduce the growth rate of *F. graminearum* hyphae [7]. These results suggest that down-regulation of transcripts related to metabolism may correlate with reduced mycelial growth in *F. graminearum*.

Ascospores and conidia are *Fusarium* head blight inocula. In the viral transmission assay, the vertical transmission rate via conidia reached 100%. Genes associated with sexual and asexual reproduction in *F. graminearum* were identified as DEGs. GO enrichment analysis showed that genes associated with conidiophore development (GO:0070787) and cell wall polysaccharide biosynthetic process (GO:0070592), were significantly down-regulated. This result was consistent with our previous study reporting a reduced spore production rate. It was worthy to note that sex-related GO terms and pathways, including meiotic chromosome segregation, centromeric DNA binding, chromosome segregation, syncytium formation by plasma membrane fusion, chromatin remodeling, mitotic sister chromatid segregation, and the spindle pole body, were enriched. All of these results suggest that FgHV1 infection caused specific transcriptome alterations and that this virus is well adapted to *F. graminearum* transmitting from generation to generation.

By binding to specific DNA sequences, transcription factors (TFs) control the rate of transcription of genetic information from DNA to mRNA [18]. There are at least 718 TFs encoded in the *F. graminearum* genome, which can be classified into 44 families [19]. Thus, differentially expressed TFs are of great interest when exploring the interaction between hypoviruses and fungi. Members of 7 of 44 TF families were found to be differentially expressed during hypovirus FgHV1 infection (Table 3). The Zn2Cys6 family (5 TFs) and C2H2 zinc fingers (5 TFs) were the most dominant TF families. Moreover, genes encoding TFs such as the bHLH, negative transcriptional regulator, nucleic acid-binding (OB-fold), bZIP, and GATA-type zinc finger TFs also showed significant changes at the transcript level. In *Fusarium* species, although the function and regulation of most TFs remain unknown, some transcription factors involved in pigmentation, mycotoxins biosynthesis, sexual development, virulence, and stress responses were investigated. For example, GIP2, a Zn2Cys6 transcription factor, regulates the expression of genes involved in the biosynthesis of the toxin aurofusarin [20]. Another type of Zn2Cys6 transcription factor present in *F. graminearum*, EBR1, is involved in the regulation of radial growth, virulence, the germination rate, and conidiation [21]. In the present study, most of the DEGs encoding Zn2Cys6 and GATA-type zinc finger TFs were observed to be down-regulated, while DEGs encoding C2H2 zinc finger and bHLH TFs were up-regulated. These differentially expressed TFs may play important roles in host-virus interactions.

**Figure 7:**
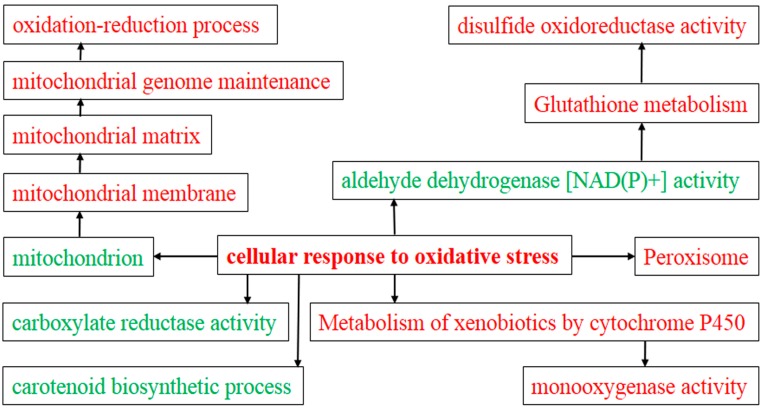
Analysis of enriched GO and KEGG terms involved in cellular redox regulation. Cellular components, molecular functions and biological process were included in the analysis. The terms that were up-regulated were colored in red and other down-regulated terms were colored in green.

Many strategies are used to constrain viral replication, including regulating the cellular redox state. In the host cell, viral RNA is recognized as nonself by the cytosolic pathogen recognition receptor, resulting in a signaling cascade relayed through the mitochondrial antiviral signaling, which is localized to both the mitochondria and peroxisomes [22]. Targeting peroxisomes is a key strategy used by viruses to subvert early antiviral defenses. Virus-induced oxidative stress can cause disruptions in normal mechanisms of cellular signaling. Whether host cells infected with hypovirus show a decrease in antioxidants has yet to be reported. In the present study, a striking number of terms involved in cellular response to oxidative stress were enriched in FgHV1-infected *F. graminearum* (Figure 7). Among the top three items identified in the GO term and pathway enrichment analyses, cell redox homeostasis (GO:0045454), protein disulfide oxidoreductase activity (GO:0015035), the cellular response to oxidative stress (GO:0034599), and metabolism of xenobiotics by cytochrome P450 (ko00980) were related to biological redox reactions. Many oxidoreductase- and glutathione-related items were also enriched. Tests confirmed that FgHV1 encoded p20 could induce H_2_O_2_ accumulation and hypersensitive response in *N. benthamiana* leaves. The *F. graminearum* host may attempt to maintain the redox balance and defend against oxidative stress under FgHV1 infection. Further research on cellular redox regulation in *F. graminearum* faced with FgHV1 infection is of great interest.

Eukaryotes can protect themselves from viral infections through RNA silencing. Viral infection can strongly induce the expression of genes involved in host RNA silencing. It has been demonstrated that the DCL2-dependent pathway is responsible for vsiRNA production and that *Ago2* is required for the induction of the RNA silencing antiviral defense in the *C. parasitica*/CHV1 system [23,24]. In *F. graminearum*, FgDicer2 is critical for sRNA transcription and micro-like RNA generation [25]. FgDicer and FgArgos transcripts were differently expressed in *F. graminearum* with and without FgHV1-infection, indicating that *F. graminearum*/FgHV1 can serve as a system for studying RNA silencing mechanisms. GO term, regulation of DNA methylation (GO:0044030), also had drawn much attention. Our previously published article showed that 24-nt small RNAs were reduced by half in FgHV1-infected *F. graminearum*, which were required to direct methylation [26]. Plant and animal viruses encode a wide variety of RNA silencing suppressors (RSSs) to resist RNA silencing [27,28]. To our knowledge, the only two RSSs identified in mycoviruses are the CHV1-encoded papain-like protease p29 and the *Rosellinia necatrix* mycoreovirus 3-encoded S10 [29,30]. Encouragingly, P20, a papain-like proteinase encoded by FgHV1, is closely related to the CHV1-encoded RSS p29, which may serve as a potential RSS and requires further investigation.

## 4. Materials and Methods

### 4.1. Fungal Strains and Culture Conditions

The *F. graminearum* strain HN10 infected with hypovirus FgHV1 and the isogenic virus-free strain HN10-11F were cultured on potato dextrose agar (PDA) (Difco, Detroit, MI, USA) at 25 °C in the dark. Liquid potato dextrose broth (PDB) cultures (50 mL) were inoculated with a plug of freshly grown mycelia (0.7 cm in diameter) and cultured for four days at 25 °C with stirring at 180 rpm in a shaker. After filtering through Miracloth (Calbiochem, San Diego, CA, USA), the hyphae were harvested, washed with distilled water twice, dried with pressure between paper towels, flash-frozen in liquid nitrogen and stored in a −80 °C freezer. To increase the accuracy of the project, three biological replicates were included for each sample.

### 4.2. Vertical Transmission Rate via Asexual Spores

Asexual spores were produced following a previously described procedure [7]. To determine the efficiency of transmission to asexual spores, the harvested spores were transferred into tubes, serially diluted and laid onto PDA plates. After 36 h, single spore cultures were removed to a new plate. Northern dot blots were conducted using RNA samples extracted from mycelia after six days of growth as described [7]. The existence or absence of viral RNA was confirmed through northern dot blotting as described above.

### 4.3. RNA Extraction and cDNA Library Construction and Sequencing

The collected hyphae were ground into powder with a grinding mill. RNA was extracted using TRIzol (Invitrogen, Carlsbad, CA, USA) according to the manufacturer’s instructions. The extracted total RNA was treated with DNase I to remove DNA contamination. The quality and concentration of extracted RNA were examined using an Agilent 2100 Bioanalyzer (Agilent Technologies, Palo Alto, CA, USA) and mRNA was isolated from the prepared RNA using Oligo (dT) magnetic beads. The isolated mRNA was mixed with fragmentation buffer and fragmented into short fragments. Then, cDNA was synthesized using the mRNA fragments as templates. Short fragments were purified and resolved with EB buffer to be used for end reparation and single-nucleotide A (adenine) addition. The short fragments were then connected with adapters. After agarose gel electrophoresis, the suitable fragments were selected as templates for PCR amplification. During the quality control (QC) steps, the Agilent 2100 Bioanalyzer and ABI StepOnePlus Real-Time PCR System (Applied Biosystems, Foster, CA, USA) were used to quantify and test the quality of the sample library. Finally, the libraries were sequenced using the Illumina HiSeq™ 2000 at BGI-Shenzhen (BGI, Shenzhen, China). For each transcriptome sample, three biological replicates were used for cDNA library construction and sequencing. The raw sequencing data have been deposited in the National Center for Biotechnology Information (NCBI) Sequence Read Archive (SRA) under accession numbers: SRA189434.

### 4.4. Identification of Differentially Expressed Genes

Raw reads produced from the Illumina HiSeq™ 2000 were filtered into clean reads and aligned to the reference sequences of *F. graminearum* PH-1 using SOAPaligner/SOAP2 [31]. The genome of the *F. graminearum* strain PH-1 (FGSC 9075, NRRL 31084) is available in the *Fusarium* comparative database (http://www.broadinstitute.org/). No more than five mismatches were allowed in the alignment. The FPKMs (Fragments per Kilobase of transcript per Million mapped reads) were calculated to determine the expression level of each gene. The differential expression analysis was performed using edgeR mRNA-seq datasets. We used a strict algorithm to identify the DEGs between two samples. DEGs were identified based on a two-fold change threshold and a false discovery rate (FDR) ≤ 0.05.

### 4.5. GO Enrichment and KEGG Analysis

According to functional categories and predefined pathways, all DEGs were analyzed. A strict algorithm based on GO-Term Finder was used to perform the GO enrichment (http://smd.stanford.edu/help/GO-TermFinder/GO_TermFinder_help.shtml/). The threshold for evaluating the significance of GO terms was obtained by applying a *p* value of 0.05. The Kyoto Encyclopedia of Genes and Genomes pathway analysis was performed in the KEGG database (http://www.genome.jp/kegg).

### 4.6. Detection of FgHV1 Induced H_2_O_2_ Accumulation and Hypersensitive Response

H_2_O_2_ and hypersensitive response induction were determined as follows. Firstly, The p20 protein of FgHV1 was purified. The p20 gene was inserted into the pET30-TEV/LIC vector (Novagen, Darmstadt, Germany). Then, the recombinant plasmid was transformed into *Escherichia coli* BL21 (DE3) (TransGen Biotech, Beijing, China). Under the optimized expression condition, overall bacterium protein was purified with a His-Trap HP column (GE Healthcare, Waukesha, WI, USA). After desalination, the concentration of purified protein was measured with the BCA kit (Pierce, Rockford, IL, USA). Purified p20 protein (5 µM) or Tris-HCL (Negative control) was injected into the *N. benthamiana* leaves using a syringe to cover areas of 1 to 2 cm^2^. The treated leaves were cut from plants after 4 h of treatment and soaked in 3,3′-diaminobenzidine (DAB)-HCl (1 mg/mL, pH 3.8) solution. After incubation for 8 h in the dark, the leaves were placed in 95% ethanol at 65 °C to remove chlorophyll and photographed. The hypersensitive response symptoms were examined during 24 h after p20 injection.

### 4.7. Real-Time RT-PCR

Reactivated mycelial plugs were placed onto a PDA plate overlaid with cellophane membranes and cultured for four days at 25 °C. Subsequently, the mycelial mass was collected from the cellophane membranes and frozen in liquid nitrogen. Total RNA was isolated using an RNA extraction kit for fungi (Transgen Biotech, Beijing, China). cDNA was synthesized with an oligo d(T) primer using a reverse transcription reagent for qPCR and diluted 1:50. Primers for the gene targets of interest were designed using Beacon Designer V8.12 and are listed in Table S4. Quantitative real-time RT-PCR (qPCR) was performed using qPCR SYBR Green mix on a CFX manager system (Bio-Rad, Hercules, CA, USA) according to the manufacturer’s instructions. The β-tubulin gene (*FGSG_09530*) was used as a reference gene to normalize the qRT-PCR results. Three independent experiments were performed.

## Figures and Tables

**Table 1:** Summary of sequencing data.

Sample	Raw Reads	Clean Reads	Clean Ratio (%) ^b^	rRNA Ratio (%) ^c^	Genome Mapping Reads	Genome Mapping Ratio (%) ^d^
HN10^1^ ^a^	47,553,250	45,550,250	95.79	0.31	42,645,244	89.68
HN10^2^ ^a^	67,251,960	61,857,850	91.98	2.71	54,383,992	80.87
HN10^3^ ^a^	59,989,576	55,086,452	91.82	1.78	48,820,879	81.38
HN10-11F^1^ ^a^	47,513,984	45,490,462	95.74	0.14	43,087,301	90.68
HN10-11F^2^ ^a^	41,710,160	39,258,412	94.12	1.20	33,089,576	79.33
HN10-11F^3^ ^a^	57,445,044	52,402,112	91.22	2.33	45,621,588	79.42

^a^ HN10^1^, HN10^2^, and HN10^3^: three biological replicates of an FgHV1-infected sample. HN10-11F^1^, HN10-11F^2^, and HN10-11F^3^: three biological replicates of an FgHV1-free sample; ^b^ Clean ratio = Clean reads/Raw reads; ^c^ rRNA ratio = rRNA/Raw Reads; ^d^ Genome mapping ratio = Genome mapping reads/Raw reads.

**Table 2:** Representative down-regulated and up-regulated *F. graminearum* genes showing relatively higher fold-changes in the FgHV1-infected strain relative to the virus-free strain.

Gene Locus	Fold_Change (Group 1/Group 2)	*p*-Value	Gene Function
**Representative down-regulated genes in FgHV1-infected strain**			
*FGSG_00226*	0.062191	9.03 × 10^−13^	Domain: Fungal trichothecene efflux pump (TRI12)
*FGSG_13962*	0.112587	1.77 × 10^−17^	Alcohol dehydrogenase
*FGSG_02138*	0.131142	4.73 × 10^−5^	Cytochrome P450 oxidoreductase
*FGSG_10802*	0.133304	1.99 × 10^−28^	Domain: LrgB-like family
*FGSG_04468*	0.144817	4.92 × 10^−10^	Domain: Transmembrane amino acid transporter protein
*FGSG_06127*	0.146473	6.70 × 10^−85^	Formate dehydrogenase
*FGSG_07582*	0.152292	0	MFS transporter
*FGSG_03984*	0.152931	1.161 × 10^−3^	l-lactate dehydrogenase
*FGSG_01450*	0.157498	6.18 × 10^−57^	Domain: Calcipressin
*FGSG_11272*	0.178327	1.05 × 10^−13^	ABC transporter
*FGSG_07598*	0.183762	2.45 × 10^−32^	Domain: Alcohol acetyltransferase
*FGSG_07509*	0.199109	5.39 × 10^−6^	Domain: Major Facilitator Superfamily
*FGSG_09697*	0.202726	5.33 × 10^−226^	Domain: multidrug resistance protein
*FGSG_10920*	0.211498	3.28 × 10^−12^	Domain: Zinc-binding dehydrogenase
*FGSG_11271*	0.222594	3.45 × 10^−4^	Domain: Fungal specific transcription factor
**Representative up-regulated genes in FgHV1-infected strain**			
*FGSG_11202*	19.12455	9.96 × 10^−13^	Domain: Guanylate kinase
*FGSG_01766*	31.36052	2.99 × 10^−41^	Branched-chain amino acid aminotransferase
*FGSG_02809*	39.84652	6.84 × 10^−182^	Domain: SRR1
*FGSG_11457*	48.10646	9.17 × 10^−137^	Endoribonuclease L-PSP
*FGSG_04616*	56.20855	1.91 × 10^−4^	Glutathione-dependent formaldehyde-activating enzyme

**Table 3:** Transcriptional factors from *F. graminearum* showing different expression levels in FgHV1-infected strain relative to virus-free strain.

Gene Locus	Fold_Change (Group 1/Group 2)	*p*-Value	Transcription Factor Family
*FGSG_11271*	0.222594	3.44 × 10^−4^	Zn2Cys6
*FGSG_03292*	0.290465	3.15 × 10^−4^	Zn2Cys6
*FGSG_04554*	0.295730	0	Nucleic acid-binding, OB-fold
*FGSG_06503*	0.324989	1.20 × 10^−4^	Zn2Cys6
*FGSG_09001*	0.335331	4.02 × 10^−6^	bZIP
*FGSG_07583*	0.367170	3.21 × 10^−4^	Negative transcriptional regulator
*FGSG_07192*	0.396762	1.71 × 10^−8^	Zn2Cys6
*FGSG_04626*	0.405624	4.77 × 10^−5^	GATA type zinc finger
*FGSG_04288*	2.109185	2.43 × 10^−42^	C2H2 zinc finger
*FGSG_12742*	2.715052	6.74 × 10^−5^	Zn2Cys6
*FGSG_02516*	2.825014	9.17 × 10^−306^	bHLH
*FGSG_10360*	3.148453	4.11 × 10^−6^	C2H2 zinc finger
*FGSG_09991*	3.360416	3.57 × 10^−7^	Nucleic acid-binding, OB-fold
*FGSG_05567*	3.933166	1.58 × 10^−30^	bHLH
*FGSG_00764*	4.940559	1.712 × 10^−3^	C2H2 zinc finger
*FGSG_09832*	5.399239	4.23 × 10^−20^	bZIP
*FGSG_04209*	7.590574	2.44 × 10^−4^	Negative transcriptional regulator
*FGSG_03881*	7.804580	1.49 × 10^−4^	C2H2 zinc finger
*FGSG_08246*	28.29656	5.34 × 10^−23^	C2H2 zinc finger

## Supplementary Materials

Supplementary materials can be found at www.mdpi.com/1422-0067/17/11/1922/s1.
