# Supplementary material for: Transcriptome-Based Discovery of Fusarium graminearum Stress Responses to FgHV1 Infection
Source: Int J Mol Sci. 2016 Nov 17;17(11):1922. doi: 10.3390/ijms17111922 (PMC5133918; doi:10.3390/ijms17111922)
Supplement: Supplementary file 1 [file ijms-17-01922-s001.zip › ijms-152770-Supplementary Materials/ijms-152770-supple-Figure S1, Table S4.pdf]

Supplementary Materials: Transcriptome-Based Discovery of *Fusarium graminearum* Stress Responses to FgHV1 Infection

Shuangchao Wang, Jingze Zhang, Pengfei Li, Dewen Qiu and Lihua Guo

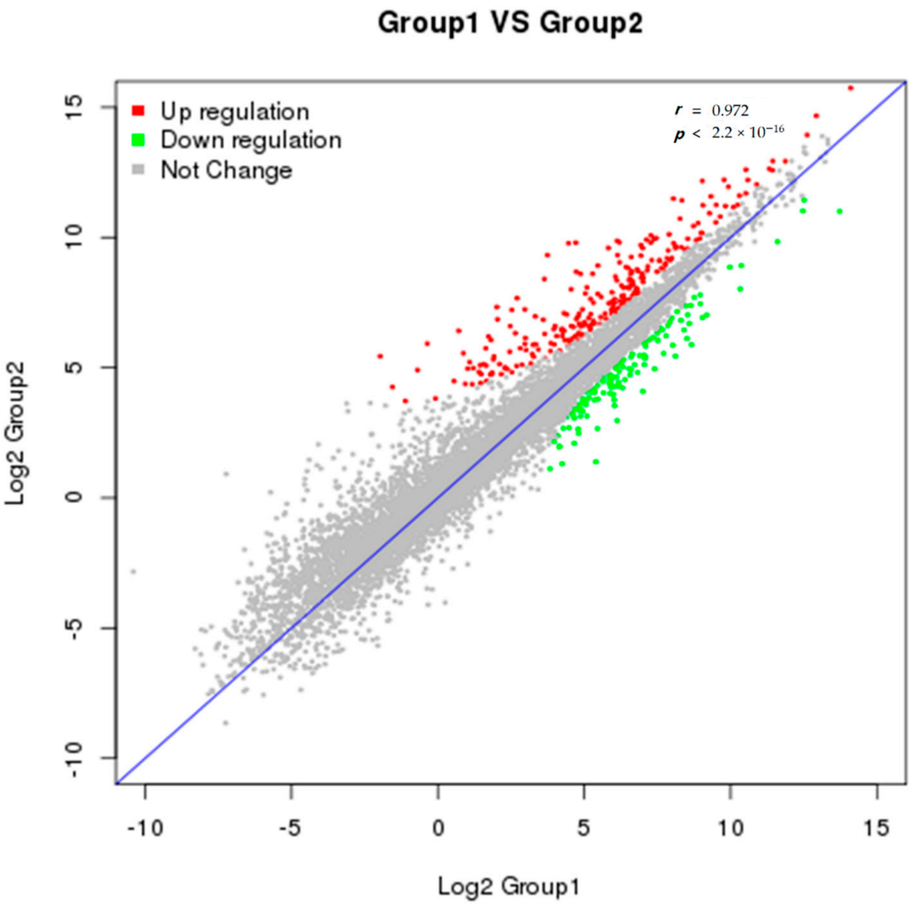

Figure S1. Correlation of the RPKM distribution between two groups. The red (Up) and green (down) dots indicate genes showing significant differences (*FDR* ≤ 0.05, at least a two-fold difference), and the gray dots indicate genes not showing significant differences. Group 1: virus-infected samples; Group 2: virus-free samples. Pearson's correlation coefficients between the two groups are presented.

Table S4. List of primers used for qRT-PCR designed by Beacon Designer V8.12 with default parameters for SYBR Green design.

**Primer**	**Sequence (5′-3′)**
FGSG_00226-F	GGTGCTCTTTGCGTAACAT
FGSG_00226-R	GGTGGCGACAATTTGAGA
FGSG_02168-F	AGACGGAAGAGCAATATGT
FGSG_02168-R	CCAATGACGTTCCAGAGA
FGSG_08700-F	CAAGGGAAACAAATACCA
FGSG_08700-R	TATAGAAGCGATGTAAGGA
FGSG_01151-F	GATACACAGGAGAGGATG
FGSG_01151-R	TCATAAGAGACTGAATAGCA
FGSG_05554-F	CGACCTATGCTCATCTTC
FGSG_05554-R	TTACTGCTTGACAATCTTCT
FGSG_03046-F	GACTGAATATGTCACCAA
FGSG_03046-R	GCTTCCACTTGTACTTAG
FGSG_03385-F	GAACTCAAGGATGGTCTC
FGSG_03385-R	CTATTCAATAACAATGCGATGA
FGSG_10543-F	TTCTTGTCTTCACCTCTT
FGSG_10543-R	TAGCACTTCTCGTATTGT
FGSG_07673-F	TGTATCATCTAGTGGAAT
FGSG_07673-R	ATATCATTGGCAAGTAAG
FGSG_07386-F	CAGGTGTAGAAGATGGAG
FGSG_07386-R	CACTACCACTACTGTCTC
FGSG_09530-F	ATGCGCGAGATTGTTCAC
FGSG_09530-F	AGAGATGGTCTGCCAGAA
